# Experience of finding footwear and factors contributing to footwear choice in people with gout: a mixed methods study using a web-based survey

**DOI:** 10.1186/s13047-018-0313-y

**Published:** 2019-01-08

**Authors:** Angela Brenton-Rule, Nicola Dalbeth, N. Lawrence Edwards, Keith Rome

**Affiliations:** 1 Department of Podiatry, Health and Rehabilitation Research Institute, Auckland University of Technology, Private Bag 92006, Auckland, 1142 New Zealand; 2 Faculty of Medical and Health Sciences, The University of Auckland, Private Bag 92019, Auckland, 1142 New Zealand; 3 Department of Rheumatology, Auckland District Health Board, P.O. Box 92189, Auckland, New Zealand; 4 Department of Medicine, University of Florida, Gainesville, USA

**Keywords:** Gout, Footwear, Shoes

## Abstract

**Background:**

Gout frequently affects the foot, particularly the first metatarsophalangeal joint. People with gout commonly wear ill-fitting footwear that lacks cushioning and support, which may further contribute to foot pain and disability. Footwear with good cushioning and motion control may be an effective non-pharmacological intervention. Currently, there is limited understanding about the footwear experience in people with gout. The aim was to understand footwear characteristics, experience of finding footwear, and factors contributing to footwear choice, in people with gout.

**Methods:**

A web-based survey of people visiting a gout education website. Participants self-reported a diagnosis of gout. The 17-item survey included questions to elicit demographic and clinical characteristics, type of footwear worn, level of difficulty finding appropriate footwear, and factors contributing to choices about footwear. A mixed quantitative and qualitative methodology was used to report survey findings.

**Results:**

Survey respondents (*n* = 83) were predominately White/Caucasian (84%), male (58%), and aged between 46 and 75 years-old (73%). Thirty-nine percent were newly diagnosed (< 12 months), 43% had gout for 1–10 years, and 19% had disease over 10 years. Gout flares in the feet were reported by 77 (93%) respondents, mostly in the big toe joint (73%). Seventy-six (92%) participants completed questions about footwear. Closed-in athletic shoes (88%), sturdy walking shoes (79%), and casual closed-in slip-on shoes (63%) were most frequently worn. Orthopaedic shoes were worn least often (16%). Comfort, fit, support, and ease to put on/take off were the features most often rated as important or very important when choosing footwear. Over half the respondents (64%) reported difficulty in finding footwear. Three categories, encompassing seven subcategories, were identified from the qualitative analysis to describe experiences of footwear. Categories included difficulty finding suitable shoes; impact of shoes on activity; and preferred footwear.

**Conclusions:**

People with gout need comfortable shoes that conform to the foot, have a wide opening, made from pliable materials with adjustable straps. The main barriers related to footwear include difficulty finding shoes that are wide enough, suitable for work and aesthetically pleasing. These findings provide clinicians with important insights into the priorities and needs of people with gout that should be considered when developing footwear interventions.

## Background

Gout is one of the most common and painful forms of inflammatory arthritis affecting over 3% of adults in developed countries [1]. The central pathogenic feature of gout is deposition of monosodium urate crystals in and around the joints [2]. Clinical manifestations include recurrent gout flares, chronic gouty arthritis, and tophi leading to progressive joint damage and reduced quality of life [3]. Gout frequently affects the feet, particularly the first metatarsophalangeal joint, but also the midfoot and in advanced disease can affect the Achilles tendon [4]. Therefore, gout is of particular importance and relevance to health care professionals who manage foot problems. The impact of gout on the ability to find appropriate footwear is often overlooked [5], as is the influence of footwear on disease management and quality of life [6].

Footwear is routinely used as a non-pharmacological intervention in people with foot and ankle arthritis [7]. However, a previous study conducted by our group found that people with gout frequently experience problems relating to footwear [8]. In addition, we found that the use of poor footwear was common in people with gout and was associated with foot disability and impairment [8]. Poor footwear characteristics included poor cushioning, lack of support, lack of stability and motion control. Important factors in patients’ choice of footwear included comfort, fit, support and cost [8]. A subsequent feasibility study demonstrated significant improvements in foot-related pain and disability when footwear with good cushioning and motion control were worn [6].

Understanding how people with gout experience footwear may help clinicians to adopt a more patient centred approach in the provision of footwear as a therapeutic intervention [9, 10]. Currently, there is limited understanding about the footwear experience in people with gout and what factors contribute to their footwear choice. A qualitative study exploring people’s knowledge of gout described the inability to put shoes on, or wear appropriate shoes, as impacting on work and social life [5]. In addition, our group explored experiences of footwear in people with inflammatory arthritis, including a small number with gout, using a web-based survey [11, 12]. Issues relating to footwear in people with inflammatory arthritis included difficulty in finding appropriate footwear, dissatisfaction with prescribed therapeutic footwear and the high cost of footwear [11, 12]. Further work is required to gain a deeper understanding around issues of footwear faced in people with gout. The aim of this study was to understand footwear characteristics, experience of finding appropriate footwear, and factors contributing to decisions about footwear choice, in people with gout.

## Methods

A cross-sectional observational study using a web-based survey. The survey was promoted by the Gout and Uric Acid Education Society (GUAES) via the GUAES website and Facebook page. The GUAES is a non-profit organization dedicated to raising the awareness of gout and the provision of educational resources to the public and health professionals. Participants were a convenience sample of adults who accessed the GUAES website and self-identified as having gout. Ethical approval was obtained from the Auckland University of Technology Ethics Committee (AUTEC 16/75). The survey was anonymous and self-administered. Consent was obtained via submission of the completed survey.

The survey was developed and tested by all co-authors. Survey questions were initially developed from our previous work in people with inflammatory arthritis [11] and revisions were made to ensure the relevance to people with gout. The survey comprised of 17 questions and was pilot-tested by people with gout and all co-authors. Revisions were based on patient feedback, previous research [5, 6, 8] and clinical experience. All co-authors agreed on the final version of the survey. Questions 1–3 were to obtain demographic data including gender, age and ethnicity. Questions 4–11 sought to elicit information relating to the participants’ gout including disease duration, frequency and location of flares, medications and current foot pain. Question 12 was designed to determine the respondents’ overall level of difficulty in finding appropriate footwear. Question 13 elicited information related to the current footwear styles worn by the participant. A list of 6 styles of footwear was provided, with the addition of barefoot and socks. Styles included closed-in athletic shoes or sneakers, sports-style supportive sandals, fashion sandals or flip-flops, casual closed-in slip-on shoes, sturdy walking shoes or boots and orthopedic or customized shoes [13]. Participants were asked to rate how often they wear each footwear type (Never, Sometimes, Mostly, Always). Question 14 sought to elicit participant reports of the most important features when choosing footwear. The presented response options (Not important, Slightly important, Of importance and Very important) were based upon previous studies [14, 15]. Questions 15–17 were open-ended questions that sought to elicit participants’ thoughts, opinions and feelings on their experience of footwear including barriers related to footwear and the impact of footwear on the ability to do the things they want to do. The online software, Survey Monkey http://www.surveymonkey.com, was utilized for the survey [16, 17]. A hyperlink to the survey was placed on the GUAES website http://gouteducation.org/. The survey was open between March 2016 and October 2017.

The method included the collection of both quantitative and qualitative data. The primary analysis was descriptive statistics to summarize quantitative survey results. Responses to questions 1–14 were collated by the Survey Monkey software and manually transferred into tables. Responses to qualitative questions relating to experiences of footwear (questions 15–17) were consecutively compiled in an unstructured transcript document. A conventional content analysis approach was adopted to analyse the open-ended responses. This approach aims to describe a phenomenon, in this case experiences of footwear, where research on the area is limited. The goal of content analysis is to classify large amount of text into categories that represent similar meaning [18]. In conventional content analysis, the researcher avoids using preconceived categories, instead allowing new insights to be developed [18]. Data coding was inductive or data-led, meaning that the data itself was the starting point for analysis. The qualitative analysis was conducted by one researcher (ABR).

## Results

### Demographic and clinical features

Table 1 summarizes the demographic and clinical characteristics of the participants. Eighty-three people responded to the survey. Survey respondents were predominantly White/Caucasian (*n* = 70, 84%), male (*n* = 48, 58%) and aged between 46 and 75 years old (*n* = 61, 73%). Participants represented a diverse cross-section of disease duration with 39% (*n* = 32) newly diagnosed (< 12 months), 43% (*n* = 35) with gout for 1–10 years and 19% (*n* = 16) with longstanding disease (> 10 years). Eighty-one percent (*n* = 67) of participants reported at least one flare in the past three months. Gout flares in the feet were common (*n* = 77, 93%) and mostly in the big toe joint (*n* = 59, 73%). Over one third (*n* = 29, 35%) reported tophi in the feet.

**Table 1:** Demographic and clinical characteristics

Male gender, n (%)	48 (58)
Age group, n (%)	
16–25 years	2 (2)
26–35 years	7 (8)
36–45 years	7 (8)
46–55 years	17 (20)
56–65 years	23 (28)
66–75 years	21 (25)
76 years or older	6 (7)
Ethnicity, n (%)	
American Indian or Alaskan Native	1 (1)
Asian	4 (5)
Black or African American	2 (2)
Hispanic	4 (5)
White/Caucasian	70 (84)
Native Hawaiian or other Pacific Islander	1 (1)
Māori	1 (1)
Gout disease duration, n (%)	
Less than 6 months	22 (27)
6 months to 1 year	10 (12)
1–5 years	22 (27)
6–10 years	13 (16)
11–15 years	5 (6)
More than 15 years	11 (13)
Number of gout flares in past 3 months, n (%)	
None	16 (19)
1–2	47 (57)
3 or more	20 (24)
Gout affecting the feet, n (%)	77 (93)
Tophi affecting the feet, n (%)	29 (35)
Location of gout flares in the feet, n (%)	
Big toe joint	59 (73)
Big toe	20 (25)
Lesser toes	18 (22)
Midfoot	21 (26)
Ankle	26 (32)
Heel	8 (10)
Other	10 (12)
Current foot pain, n (%)	73 (88)
Gout medications, n (%)	
Non-steroidal anti-inflammatory drugs	27 (33)
Corticosteroids	9 (11)
Colchicine	24 (29)
Allopurinol	34 (41)
Febuxostat	5 (6)
No medication	8 (10)

### Footwear questions

Seventy-six participants (92%) responded to questions relating to footwear. Table 2 summarizes the footwear worn by respondents. Closed-in athletic shoes (*n* = 66, 87%), sturdy walking shoes or boots (*n* = 60, 79%) and casual closed-in slip-on shoes (*n* = 48, 63%) were reported as the most frequently worn footwear type. Over one quarter mostly or always wore socks or bare feet only. Orthopedic or customized shoes (*n* = 12, 16%) were reported as worn least often. Table 3 summarizes the features of importance in choosing footwear. Comfort (*n* = 74, 97%), fit (*n* = 75, 99%), support (n = 60, 79%) and ease to put on/take off (*n* = 57, 75%) were the features most often rated as important or very important when choosing footwear. Footwear material (*n* = 29, 38%) and fastenings (*n* = 28, 37%) were reported as the least important footwear attributes.

**Table 2:** Type of footwear worn by respondents

Footwear worn	Never	Sometimes	Mostly	Always
Closed-in athletic shoes/sneakers	10 (13)	30 (40)	34 (45)	2 (3)
Sturdy walking shoes or boots	16 (21)	35 (46)	19 (25)	6 (8)
Casual closed-in slip-on shoes	28 (37)	29 (38)	14 (18)	5 (7)
Bare feet or socks only	32 (42)	23 (30)	17 (22)	4 (5)
Fashion sandals/flip flops	38 (50)	30 (39)	6 (8)	2 (3)
Sport-style supportive sandals	43 (57)	26 (34)	4 (5)	3 (4)
Orthopedic or customized shoes	64 (84)	6 (8)	3 (4)	3 (4)

**Table 3:** Features of importance in choosing footwear

Features of importance	Not important	Slightly important	Of importance	Very important
Comfort	1 (1)	1 (1)	13 (17)	61 (80)
Fit	0 (0)	1 (1)	22 (29)	53 (70)
Support	4 (5)	12 (16)	21 (28)	39 (51)
Ease to put on/take off	2 (3)	17 (22)	27 (36)	30 (39)
Heel height	23 (30)	19 (25)	11 (14)	23 (30)
Cost	10 (13)	20 (26)	29 (38)	17 (22)
Non-slip	11 (14)	22 (29)	29 (38)	14 (18)
Style	8 (11)	30 (39)	28 (37)	10 (13)
Weight	9 (12)	34 (45)	27 (36)	6 (8)
Color	19 (25)	23 (30)	28 (37)	6 (8)
Material	14 (18)	33 (43)	21 (28)	8 (11)
Fastenings	21 (27)	27 (36)	19 (25)	9 (12)

The majority of participants (*n* = 49, 64%) agreed or strongly agreed with the statement, “I have a lot of trouble finding shoes I like; that are comfortable and that I can get my foot into”.

### Qualitative analysis

There were 56 useable responses to question 15, “What barriers have you experienced related to shoes?” Forty-nine useable responses were recorded for question 16, “What impact has shoes had on your feet and your ability to do the things you wanted to do?” and 27 for question 17, “Is there anything else you would like to share about your experience with shoes and foot problems in gout?” Three categories, encompassing seven subcategories, were identified from the qualitative data analysis: difficulty finding suitable shoes; impact of shoes on activity; and preferred footwear. Illustrative quotes from participant responses have been selected to represent each subcategory.

#### Difficulty finding suitable shoes

The responses describing difficulty in finding suitable shoes were grouped into three subcategories relating to comfort/fit, style and cost. The majority of participants described difficulty in finding shoes that are comfortable and fit well particularly during a gout flare. A number of respondents described their shoes as hurting their feet or exacerbating swelling. Many admitted to not being able to wear any shoes during a flare or said it was difficult or impossible to find shoes that did not hurt their feet.

*“Going out my feet kill me when I have shoes on.”* Male, American Indian/Alaskan Native, 46-55, gout <6 months.

*“Some shoes make the swelling worse and other shoes aren’t padded enough.”* Female, White, 26-35, gout <6 months.

*“It’s impossible to stuff my foot in any shoe during a flare up. I resort to a sock.”* Female, White, 46-55, gout <6 months.

Lack of width was the predominant concern with most participants describing experiences of shoes that are too narrow to get into, or too tight for their wide feet. Shoes were also described as being too rigid or heavy, lacking support, and having padding in the wrong places.

*“Being wide enough. Being able to “open them up” enough to get them on.”* Female, White, 46-55, gout <6 months.

*“My feet are very short and wide, now with gout my foot is even wider. I had trouble finding shoes before now it is almost impossible to find shoes that fit.”* Female, White, 56-65, gout 1-5 years.

Issues with ill-fitting shoes were also described, such as shoes wide enough to accommodate the forefoot that then slipped at the back. One participant described buying dress shoes a size larger, in case of a flare. A further problem was asymmetry in foot shape with one wide and one ‘normal’ foot making shoe sizing difficult.

*“I hate having my toes crowded together. So often I end up with shoes that are comfortable around my feet but then slip when I walk creating blisters.”* Female, Māori, 46-55, gout <6 months.

*“Bunion make certain styles uncomfortable, bunion make one foot wide, other foot remain average.”* Female, White, 66-75, gout 1-5 years.

Issues relating to style included difficulty in finding desirable shoes as well as shoes appropriate for work. Participants expressed frustration in finding dress shoes that are comfortable and aesthetically pleasing. Many highlighted lack of choice in a wide fitting shoe, in particular women’s shoes. Many women expressed sadness at not being able to be dressed up, wear fashionable shoes or high heels even on a special occasion. Difficulty was also expressed with finding shoes to go with different styles of clothing.

*“I enjoy wearing fashionable shoes because enjoy dressing up, but usually orthopaedic shoe are not very fashion forward.”* Male, White, 36-35, gout <6 months.

*“Pretty, easy shoes are not made for very wide feet, just ugly, painful shoes.”* Female, White, 66-75, gout 1-5 years.

Issues relating to work shoes highlighted the need for protective footwear, such as work boots, which are comfortable and light, and dress shoes that are padded and can be worn all day.

*“I need study boots for working within the Landscape company I own and heavy boots are not always the most comfortable for a gout sufferer.”* Male, White, 36-45, gout 6-10 years.

*“It’s very hard to find dress up ladies shoes that are comfy, padded, and can be worn the whole work shift.”* Female, White, 26-35, gout <6 months.

Price was a barrier for some, with some respondents expressing concern over the cost of shoes. Respondents expressed the belief that good quality shoes, suitable for a person with gout, were too expensive.

*“There has to be a reasonably priced shoe out there for gout sufferers. HELP US!”* Female, White, 56-65, gout 1-5 years.

*“I have had to buy more expensive shoes than I used to just to ensure that it is the gout flare causing the pain and not bad shoes. Yet I still struggle with keeping good shoes that work for me.”* Male, White, 36-45, gout >15 years.

#### Impact of shoes on activity

Responses concerning the impact of shoes on activity were divided into two subcategories relating to shoes impeding activity and shoes facilitating activity. Several people described their lack of comfortable footwear as affecting their ability to do the things they want to do. Difficulty in walking was also attributed to shoes that did not fit properly and many recognized that if they found the right shoes for their feet they would be more active.

*“If my shoes don’t fit properly I have difficulty walking any kind of distance.”* Male, White, 46-55, gout 1-5 years.

*“I cannot walk bare foot and I cannot find comfortable shoes. If I could find shoes wide enough for my deformed feet I could do more.”* Female, White, 56-65, gout 1-5 years.

*“Good shoes are the key to being active and doing what you want.”* Male, White, 56-65, gout <6 months.

Other comments relating to activity limitation, due to lack of footwear, included inability to work, play sport, socialize and even leave the house.

*“If I can’t put shoes on because the hurt, I usually won’t go anywhere.”* Male, White, 46-55, gout 1-5 years.

*“If I cannot walk/stand comfortably, am pretty much stuck at home or where I can go barefoot.”* Female, White, 66-75, gout 1-5 years.

#### Preferred footwear

Many respondents described the footwear features and type of footwear they preferred. Features included a wide toe box, cushioning at the back of the heel and toes, adjustable fastenings, and soft materials including leather and elastic to accommodate deformity. Preferred footwear style included shoes that support the foot and are lightweight, high boots to restrict ankle movement during a flare and open-toed sandals. Flip-flops and loose, slipper type shoes were also described by many as their preferred or only shoe option.

*“Recently I have tried a tighter shoe that is made to support/hold the foot. Very light weight and material that holds my foot is thin and not overwhelming.”* Male, White, 36-45, gout 6-10 years.

*“If I have gout in my ankle I wear high boots to stop too much movement.”* Male, Hispanic, 46-55, gout 6-10 years.

*“Slip-on shoes are the best type.”* Male, Hispanic, 46-55, gout >15 years.

*“Generally I wear Birkenstocks and they are the best for me.”* Female, White, 66-75, gout 1-5 years.

## Discussion

This mixed methods study provides new insights into the experience of finding footwear and factors contributing to decisions about footwear, in people with gout. Footwear is often seen as less of an issue for males than females. Therefore, the current findings are of particular significance when considering the known gender distribution of 3–4:1 males: females with gout [1]. Difficulty finding suitable shoes; the impact of shoes on activity; and preferred footwear, were key themes. Survey respondents placed major emphasis on the need for comfortable shoes, particularly during a gout flare, with the majority of comments referring to foot pain caused by tight or ill-fitting shoes. The concept of comfort closely related to fit and included conformity to foot shape, pliable materials, wide opening and adjustable straps.

In agreement with our previous New Zealand-based survey of people with inflammatory arthritis [11], closed-in athletic shoes and sturdy walking shoes were reported as most frequently worn. However, in the current study of people with gout, 63% of respondents also reported frequent use of casual closed-in slip-on shoes. The features of most importance when choosing footwear; comfort, fit and support, also reflect our previous work [8, 11]. In addition, ease to put on/off was also highly rated, which may reflect the popularity of casual slip-on shoes. In the current study, over half the respondents wore footwear with poor structural characteristics including casual slip-on shoes, fashion sandals and flip-flops. However, several commented that this was their preferred or only shoe option during a flare. Changing footwear needs, dependent on current disease activity, may therefore be an important consideration when advising patients on footwear. The use of footwear with poor structural and motion control properties also reflects our previous findings [8, 11, 14], and might suggest a lack of awareness of the importance of good quality footwear in reducing pain and disability.

The qualitative findings relating to footwear experiences support and add to our prior work in people with inflammatory arthritis in New Zealand [12], as well as studies in Australia [19] and the United Kingdom [9, 10, 20] in people with rheumatoid arthritis. Participant responses describing difficulty finding suitable shoes reflect those of our earlier work which identified change in foot shape, disease-related foot symptoms and lack of desirable footwear as key issues [12]. In the current study, lack of width was the predominant concern, which likely reflects the frequency of gout in the first metatarsophalangeal joint [4]. The perception that suitable footwear is too expensive and difficult to find also reflects our previous work [12].

Our findings relating to the impact of shoes on activity suggest that the inability to find shoes that fit and are comfortable significantly impact activity and quality of life. The impact of gout on lifestyle, social life and work life has been previously described [3, 5]. The current study adds to our understanding of the role of footwear in both limiting and facilitating activity. The notion of footwear causing foot pain is of importance when considering footwear as a management option. Previous work by our group demonstrated the impact of footwear on plantar pressures and gait in people with gout [21] therefore the potential for footwear to alter foot function should be carefully considered. Clinicians need to consider whether a footwear intervention might cause further pain and disability if the wrong shoe type is recommended. The findings of the current study may assist clinicians in determining the right shoe type for their patients with gout. In addition, it is important to consider that whilst good footwear is an effective intervention for foot pain, impairment and disability in people with foot and ankle arthritis including gout [7], footwear that does not fit properly may serve to increase pain and disability [8, 14]. Indeed, previous studies describe a relationship between ill-fitting or poor footwear and increased foot pain in people with gout [8] and rheumatoid arthritis [14].

The study is not without limitations. The majority of survey respondents were White/Caucasian, however, the prevalence of gout is higher in other ethnicity groups, including Black/African American and Asian people in the United States [22], indigenous New Zealanders (Māori), or Pacific people living in New Zealand [23]. In addition, the response rate of 58% male does not reflect the known gender distribution of gout [1]. Therefore, further work is required to understand the issues of footwear faced by men and other ethnicity groups who are disproportionately affected by gout. Conventional content analysis is limited in its ability to gain a complete understanding of context [18]. However, the brevity of responses elicited via a survey and inability to clarify meaning through face-to-face questioning limits the ability to determine/interpret deeper meaning. Further work is required to gain a deeper understanding around the issues of footwear faced in people with gout, and the meaning of this in the context of their whole experience of gout.

## Conclusions

This study is the first to report experiences of footwear in people with gout using a mixed methods approach. We found that people with gout need comfortable shoes that conform to the foot, have a wide opening, a wide and deep toe box so that no pressure is on the afflicted joint(s), and made from pliable materials with adjustable straps. The main barriers related to footwear include difficulty in finding shoes that are wide enough, suitable for work and aesthetically pleasing. The inability to find comfortable shoes, or wear any shoes during a flare, significantly impacts on activity. The findings of this study provide clinicians with important insights into the priorities and needs of people with gout that should be considered when developing footwear interventions. In addition, when prescribing footwear as a therapeutic intervention, clinicians should consider footwear preferences and issues of footwear both during and between gout flares.

## Abbreviations

GUAES: Gout and Uric Acid Education Society
